# Analgesic and Sedative Effects of Different Doses of Dexmedetomidine Combined with Butorphanol in Continuous Analgesia after a Cesarean Section

**DOI:** 10.3389/fsurg.2022.896536

**Published:** 2022-05-04

**Authors:** Hui Liu, Yalin Wang, Fulong Li, Wei Ren, Li Yuan

**Affiliations:** ^1^ Department of Anesthesia, The First Affiliated Hospital of Hebei North University, Zhangjiakou, China; ^2^ The Operating Room, The First Affiliated Hospital of Hebei North University, Zhangjiakou, China

**Keywords:** dexmedetomidine, butorphanol, sedative, analgesic, cesarean section

## Abstract

**Objective:**

The present study is designed to study the analgesic and sedative effect of different doses of dexmedetomidine combined with butorphanol in continuous analgesia after a cesarean section.

**Methods:**

A total of 60 puerperae undergoing a cesarean section recruited from a single center were divided into three groups according to the postoperative continuous analgesia protocol: control group (100 mL of normal saline containing 10 µg/kg fentanyl and 0.25 mg of palonosetron, 2 mL/h for continuous analgesia for 48 h), DB1 group (100 mL of normal saline containing 1.0 µg/kg dexmedetomidine, 4 mg of butorphanol, 10 µg/kg fentanyl, and 0.25 mg of palonosetron, 2 mL/h for continuous analgesia for 48 h), and DB2 group (100 mL normal saline containing 2.0 µg/kg dexmedetomidine, 4 mg of butorphanol, 10 µg/kg fentanyl, and 0.25 mg of palonosetron, 2 mL/h for continuous analgesia for 48 h). We compared the blood pressure, heart rate, oxygen saturation, VAS score, Ramsay score, and adverse reactions of puerperae among the three groups after surgery.

**Results:**

The baseline data all have no significant difference in the three groups (*p* > 0.05). Compared with those in the control group, the systolic blood pressure, diastolic blood pressure, heart rate, and VAS score of the puerperae in the DB1 group and DB2 group were significantly decreased at 6, 24, and 48 h (*P* < 0.05), while the Ramsay scores of the puerperae in DB1 group and DB2 group were significantly increased at 6, 24, and 48 h (*p* < 0.05). At the same time, the systolic blood pressure, diastolic blood pressure, heart rate, and VAS score of the puerperae in the DB2 group were significantly lower than those in the DB1 group (*P* < 0.05), while the Ramsay scores of the puerperae in DB2 group were significantly higher than those in the DB1 group (*P* < 0.05). Also, there is no significant difference in oxygen saturation and adverse reactions of puerperae among the three groups after surgery (*p* > 0.05).

**Conclusion:**

Dexmedetomidine combined with butorphanol can improve the analgesic and sedative effects in continuous analgesia after a cesarean section, and the analgesic and sedative effects of dexmedetomidine in the high-dose group are better than those in the low-dose group.

## Introduction

Postoperative pain-induced stress can lead to a series of changes in physiology, psychology, and behavior and adversely affect the normal endocrine and immune functions of humans (1, 2). Previous studies have shown that postoperative pain is closely related to the occurrence of postoperative complications, delays postoperative recovery, and affects patient prognosis (3, 4). In addition, severe postoperative pain is considered to be a high-risk factor for long-term chronic pain after surgery (5, 6). After a cesarean section, pain seriously affects the daily activities of the puerperium, and it is not conducive to early postpartum lactation and lactation and hinders the communication between the mother and the baby (7, 8). Therefore, it is of great significance to provide good, safe, and reliable analgesia to puerperae. Adequate postoperative analgesia can not only relieve postoperative fatigue and improve the comfort and satisfaction of puerperae but also help to improve the quality of postoperative recovery of puerperae (7, 8). However, there is currently no standard protocol for postcesarean section analgesia.

Dexmedetomidine is an α2 adrenergic receptor agonist with high selectivity and has sedative, analgesic, antisympathetic, anxiolytic, and stress-reducing effects, while it rarely causes respiratory depression (9–11). Butorphanol, an analgesic, exerts its analgesic effect mainly by agonizing κ receptors and partially antagonizing μ receptors (12). In addition, butorphanol is widely used in postoperative patient-controlled intravenous analgesia (PCIA), but it can cause many side effects when used alone (13). However, clinical studies suggest that dexmedetomidine can enhance the analgesic effect of butorphanol, and its combination can reduce its adverse effects (14, 15). In the present study, we designed to study the analgesic and sedative effect of different doses of dexmedetomidine combined with butorphanol in continuous analgesia after a cesarean section.

## Materials and Methods

### Ethics Statement

This study complied with the principles of the Declaration of Helsinki and was reviewed and approved by the Ethics Committee of the First Affiliated Hospital of Northern College in Hebei Province. In addition, all volunteers who participated in this study were informed about the content of this study and signed an informed consent form.

### Study Population

From January 2019 to December 2019, 60 puerperae undergoing a cesarean section were recruited in the present study. These puerperae must meet the following standards. Inclusion criteria are as follows: (1) undergoing a cesarean section under combined spinal–epidural anesthesia; (2) The American Society of Anesthesiologists (ASA) grades of grades I to II; (3) first birth, between 20 and 35 years old, with a height of 1.5–1.75 m; and (4) informed and agreed to participsate in this research. Exclusion criteria are as follows: (1) long-term use of nonsteroidal anti-inflammatory drugs, opioids, tranquilizers, and sedatives; (2) mental illness, intellectual disability, or other reasons for the maternal inability to complete the pain and sedation score; (3) preoperative heart rate below 50 bpm with abnormal cardiac conduction and rhythm; (4) neuromuscular endocrine diseases and allergic reactions to α2 adrenergic receptor agonists; (5) having a history of abdominal surgery in the past; and (6) anesthesia fails, the operation time is more than 2 h, the intraoperative blood loss is more than 500 mL, or a second operation is required to treat postpartum hemorrhage.

### Anesthesia and Postoperative Analgesia Protocol

All participants received combined spinal–epidural anesthesia, with open venous access to infuse ringer sodium lactate (state approval no. H20044239; Shandong Weigao Pharmaceutical Co., Ltd., Shandong, China) and choosing the lumbar 3–4 space as the puncture point. Ephedrine hydrochloride injection (state approval no. H22020730; Tonghua Baishan Pharmaceutical Co., Ltd., Jilin, China) or phenylephrine (state approval no. H31021175; Shanghai Hefeng Pharmaceutical Co., Ltd., Shanhai, China) is given to maintain circulatory stability according to changes in the blood pressure and heart rate.

Sixty patients were divided into three groups according to the postoperative continuous analgesia protocol: control group, DB1 group, and DB2 group. The control group received 100 mL of normal saline containing 10 µg/kg fentanyl and 0.25 mg palonosetron, 2 mL/h for continuous analgesia for 48 h. The DB1 group received 100 mL of normal saline containing 1.0 µg/kg dexmedetomidine, 4 mg butorphanol, 10 µg/kg fentanyl, and 0.25 mg palonosetron, 2 mL/h for continuous analgesia for 48 h. The DB2 group received 100 mL of normal saline containing 2.0 µg/kg dexmedetomidine, 4 mg butorphanol, 10 µg/kg fentanyl, and 0.25 mg palonosetron, 2 mL/h for continuous analgesia for 48 h.

### Data Collection

Herein, we used a visual analogue scale (VAS) score to assess pain at 6, 24, and 48 h after the cesarean section. Additionally, VAS scores range from 0 to 10, with higher scores indicating more severe pain. We used the Ramsay score to assess the level of sedation at 6, 24, and 48 h after the cesarean section, and the Ramsay scores range from 1 to 6, with higher scores indicating higher levels of sedation. We collected the clinical data of participants of age, height, weight, gestational week, onset time of operation, fetal delivery time, duration of surgery, and duration of anesthesia. At the same time, the blood pressure, heart rate, oxygen saturation, VAS score, and Ramsay score were also being determined at 1, 6, 24, and 48 h after the cesarean section. In addition, the adverse reactions including malignant vomiting, dizziness, itchy shin, bradycardia, and low blood pressure were also recorded immediately after surgery to 1 week after surgery.

### Statistical Analysis

Data in the present study were analyzed by SPSS 19.0 software (SPSS Inc., Chicago, USA). Qualitative data are presented as counts (%), and *p*-values are calculated using chi-square or Fisher’s exact test as appropriate. The Kolmogorov–Smirnov test was used to check whether quantitative data conformed to a normal distribution; data that conformed to a normal distribution were presented as mean ± standard deviation, and an unpaired Student’s *t*-test was used to compare differences and calculate *p*-values. Quantitative data that did not conform to a normal distribution were presented as the median (interquartile range), and the Mann–Whitney *U*-test was used to compare differences and calculate *p*-values. *p* < 0.05 were considered statistically significant.

## Results

### Demographic and Surgical Characteristics

The baseline data including characteristics including age, height, weight, and gestational week and surgical indicators including the onset time of operation, fetal delivery time, duration of surgery, and duration of anesthesia all have no significant difference in the three groups (*p* > 0.05) (**Table 1**).

**Table 1:** Comparison of demographic and surgical indicators among three groups (mean ± SD).

Groups/variables	Control group (*n* = 20)	DB1 group (*n* = 20)	DB2 group (*n* = 20)	*p-*Value
Age (years)	29.4 ± 5.8	30.5 ± 6.2	30.7 ± 5.9	0.672
Height (cm)	161.8 ± 9.2	162.0 ± 10.5	161.7 ± 9.5	0.135
Weight (kg)	70.5 ± 10.8	68.9 ± 10.2	69.7 ± 10.2	0.415
Gestational week (weeks)	37.5 ± 1.9	38.3 ± 1.5	38.1 ± 1.8	0.223
Onset time of operation (min)	16.5 ± 3.8	16.3 ± 3.7	16.2 ± 3.5	0.527
Fetal delivery time (min)	23.8 ± 3.5	23.9 ± 5.1	23.7 ± 4.8	0.408
Duration of surgery (min)	63.5 ± 9.2	60.5 ± 10.2	62.3 ±	0.411
Duration of anesthesia (min)	80.5 ± 11.2	77.9 ± 13.2	79.8 ± 10.7	0.302

### Postoperative Blood Pressure, Heart Rate, and Oxygen Saturation

At 1, 6, 24, and 48 h after the cesarean section, the systolic blood pressure (**Table 2**), diastolic blood pressure (**Table 3**), and heart rate (**Table 4**) of puerperae in the DB1 group and DB2 group are all significantly lower than those in the control group (*p* < 0.05), and the systolic blood pressure, diastolic blood pressure, and heart rate of pirpera in the DB2 group are all significantly lower than those in the DB1 group (*p* < 0.05). However, there is no significant difference among these three groups in oxygen saturation of puerpera (*p* > 0.05) (**Table 5**).

**Table 2:** Comparison of systolic blood pressure of puerperae at different times after surgery among three groups (mean ± SD, mmHg).

Group	*n*	Postoperative time (h)			
		1	6	24	48
Control group	20	126.9 ± 11.2	133.8 ± 8.9	123.6 ± 10.2	117.8 ± 8.9
DB1 group	20	120.5 ± 13.2*	123.8 ± 13.5*	117.4 ± 12.3*	114.7 ± 11.2*
DB2 group	20	110.3 ± 10.5*^#^	108.3 ± 8.7*^#^	110.2 ± 8.9*^#^	110.5 ± 10.8*^#^
*F*		7.628	13.125	8.127	4.326
*p*		<0.001	<0.001	<0.001	0.041

Compared with the control group, *p < 0.05; compared with the DB1 group, ^#^p < 0.05.

**Table 3:** Comparison of diastolic blood pressure of puerperae at different times after surgery among three groups (mean ± SD, mmHg).

Group	*n*	Postoperative time (h)			
		1	6	24	48
Control group	20	72.3 ± 8.6	78.8 ± 9.3	71.8 ± 7.6	69.2 ± 5.3
DB1 group	20	68.3 ± 7.3*	69.2 ± 6.3*	69.5 ± 4.7*	69.2 ± 4.4*
DB2 group	20	67.8 ± 6.8*^#^	68.3 ± 6.7*^#^	67.1 ± 5.6*^#^	67.2 ± 5.5*^#^
*F*		4.932	7.921	5.031	3.729
*p*		0.038	<0.001	0.021	0.048

Compared with the control group, *p < 0.05; compared with the DB1 group, ^#^p < 0.05.

**Table 4:** Comparison of the heart rate of puerperae at different times after surgery among three groups (mean ± SD, *n*/min).

Group	*n*	Postoperative time (h)			
		1	6	24	48
Control group	20	73.6 ± 8.2	81.5 ± 9.2	80.4 ± 9.1	84.2 ± 7.5
DB1 group	20	70.3 ± 8.0*	76.6 ± 9.4*	76.8 ± 9.2*	76.6 ± 6.5*
DB2 group	20	67.8 ± 6.5*^#^	65.4 ± 5.8*^#^	66.9 ± 5.7*^#^	66.3 ± 6.2*^#^
*F*		8.159	9.426	13.082	10.328
*p*		<0.001	<0.001	<0.001	<0.001

Compared with the control group, *p < 0.05; compared with the DB1 group, ^#^p < 0.05.

**Table 5:** Comparison of oxygen saturation of puerperae at different times after surgery among three groups (mean ± SD, %).

Group	*n*	Postoperative time (h)			
		1	6	24	48
Control group	20	98.5 ± 1.4	97.8 ± 1.1	98.0 ± 0.9	98.2 ± 0.7
DB1 group	20	98.3 ± 1.3	97.5 ± 1.0	97.8 ± 0.9	98.0 ± 0.8
DB2 group	20	98.4 ± 1.5	97.8 ± 1.3	98.1 ± 1.1	98.0 ± 0.8
*F*		0.841	0.921	0.756	0.792
*p*		0.372	0.264	0.492	0.415

### VAS Score for the Analgesic Effect

The VAS scores of puerperae at 6, 24, and 48 h after the cesarean section in the DB1 group and DB2 group are all significantly lower than those in the control group (*P* < 0.05), and the VAS scores of puerperae at 6, 24, and 48 h after the cesarean section in the DB2 group are significantly lower than those in the DB1 group (*P* < 0.05) (**Table 6**).

**Table 6:** Comparison of VAS scores of puerperae at different times after surgery among three groups (mean ± SD, score).

Group	*n*	Postoperative time (h)		
		6	24	48
Control group	20	3.1 ± 0.8	2.5 ± 0.4	1.9 ± 0.6
DB1 group	20	2.6 ± 0.6*	1.8 ± 0.6*	1.4 ± 0.7*
DB2 group	20	1.8 ± 1.0*^#^	1.4 ± 0.6*^#^	1.1 ± 0.4*^#^
*F*		6.829	13.218	10.081
*p*		<0.001	<0.001	<0.001

Compared with the control group, *p < 0.05; compared with the DB1 group, ^#^p < 0.05.

### Ramsay Score for the Calming Effect

The Ramsay scores of puerperae at 6, 24, and 48 h after the cesarean section in the DB1 group and DB2 group are all significantly higher than those in the control group (*p* < 0.05), and the Ramsay scores of puerperae at 6, 24, and 48 h after the cesarean section in the DB2 group are significantly higher than those in the DB1 group (*p* < 0.05) (**Table 7**).

**Table 7:** Comparison of Ramsay scores of puerperae at different times after surgery among three groups (mean ± SD, score).

Group	*n*	Postoperative time (h)		
		6	24	48
Control group	20	2.1 ± 0.6	2.6 ± 0.5	2.3 ± 0.3
DB1 group	20	2.6 ± 0.6*	3.1 ± 0.4*	2.6 ± 0.5*
DB2 group	20	2.9 ± 0.0*^#^	3.4 ± 0.5*^#^	2.8 ± 0.3*^#^
*F*		4.439	8.942	4.908
*p*		0.039	<0.001	0.035

Compared with the control group, *p < 0.05; compared with the DB1 group, ^#^p < 0.05.

### Adverse Reactions

There is no significant difference among the control group, DB1 group, and DB2 group in the adverse reactions at 48 h after surgery (*p* > 0.05) (**Table 8**).

**Table 8:** Comparison of adverse reactions at 48 h after surgery among three groups (*n*, %).

Group	*N*	Malignant vomiting	Dizziness	Itchy shin	Bradycardia	Low blood pressure	Total
Control group	20	3 (15.0)	0 (0.0)	2 (10.0)	0 (0.0)	0 (0.0)	5 (25.0)
DB1 group	20	1 (5.0)	0 (0.0)	0 (0.0)	1 (5.0)	0 (0.0)	2 (10.0)
DB2 group	20	1 (5.0)	0 (0.0)	0 (0.0)	1 (5.0)	1 (5.0)	3 (15.0)

## Discussion

In this study, we found that continuous intravenous infusion of dexmedetomidine along with butorphanol in PCIA after a cesarean section could lead not only to pain reduction and enhanced analgesic effect (according to VAS) but also improved sedative effects, while it did not affect the adverse reaction. At the same time, we also found that high-dose dexmedetomidine was more effective for analgesia and sedation than low-dose dexmedetomidine.

The use of opioids alone for sedation after a cesarean section has many disadvantages. On the one hand, the analgesic effect is insufficient (16). On the other hand, the application of high-dose opioid analgesics significantly increased the incidence of postoperative nausea, vomiting, and respiratory depression (17). In addition, the safety of many analgesics excreted in breast milk in newborns is uncertain (16, 17). Dexmedetomidine is a nonopioid drug with both peripheral and central analgesic effects, and it is a selective α2 adrenoceptor agonist, which can reduce sympathetic nerve activity, the release of catecholamines, and the harmful stimulation caused by surgery and has a certain protective effect on important organs. Results from a randomized and placebo-controlled study have shown that the combination of sufentanil and dexmedetomidine for PCA after a cesarean section can reduce sufentanil consumption and improve parturients’ satisfaction compared with sufentanil PCA alone (18). In addition, a recent study indicated that adding dexmedetomidine to the postoperative analgesia regimen for a cesarean section could enhance postoperative analgesia and improve satisfaction (19). In previous studies (20–22), the doses of dexmedetomidine for sustained analgesia after a cesarean section included 0.08, 0.5, and 3 µg/kg/h, so we administered 1.0 and 2.0 µg/kg/h dexmedetomidine to determine the dose-dependent effect and optimal dose of analgesic management with PCIA in the present study.

Furthermore, cesarean section-induced pain is not only from incision but also from uterine contraction during uterine involution, and there is no evidence suggesting that opioids are effective for uterine contraction pain (16). Butorphanol exerts an analgesic effect through the dual action of opioid receptor agonism-antagonism, and its single application has a definite effect on the treatment of severe and moderate pain (12, 13). More importantly, butorphanol can also relieve visceral pain, which can make up for the lack of opioid analgesics in postoperative analgesia for mothers (23, 24). In this study, we found that maternal addition of butorphanol for analgesia was effective in reducing uterine cramping pain, suggesting that butorphanol can relieve visceral pain in postcesarean section analgesia. Postoperative pain is the main factor leading to postoperative sleep deprivation, and patients with severe sleep deprivation are prone to postoperative hyperalgesia (25, 26). In addition, butorphanol has many advantages for PCIA after a cesarean section because of its unique pharmacological properties. First, the analgesic mechanism of butorphanol is mainly achieved by activating κ receptors, and the activation of κ receptors enables it to exert a more potent analgesic effect in the treatment of visceral pain (23, 24). Second, the milk secretion rate of butorphanol is low, and the drug concentration in maternal milk at therapeutic doses is extremely low, so there is almost no obvious adverse effect on the newborn (27).

Dexmedetomidine acts on the α2 adrenoceptor agonist in the nucleus locus coeruleus, reduces the release of norepinephrine, simulates natural sleep, and produces sedative, hypnotic, and anxiolytic effects (28, 29). In the present study, we found that dexmedetomidine combined with butorphanol can improve the sedative effects (according to the Ramsay score) in continuous analgesia after a cesarean section, and the analgesic and sedative effects of dexmedetomidine in the high-dose group are better than those in the low-dose group, which is consistent with the findings of Zhang et al. (30). Zhang et al. found that dexmedetomidine was more effective than sufentanil for maternal labor sedation, and the analgesic and sedative effects of dexmedetomidine in the high-dose group were better than those in the low-dose group (30).

Postoperative pain can induce adverse events, such as cardiovascular disease, respiratory depression, digestive and urinary system dysfunction, and neuroendocrine disorders, which bring great discomfort to patients and seriously affect the postoperative recovery of patients (31, 32). At the same time, although the use of unreasonable postoperative analgesics can effectively control pain, it can also cause adverse reactions, such as malignant vomiting, dizziness, itchy skin, bradycardia, and low blood pressure (33, 34). Therefore, to date, there is still no accepted and optimal analgesic regimen for postoperative pain. In our study, we found that the addition of dexmedetomidine combined with butorphanol to the basic postoperative analgesia regimen enhanced the analgesic effect without increasing the adverse reactions in patients, which suggested that dexmedetomidine combined with butorphanol is not only effective in postoperative analgesia and sedation but also safe in puerperae after a cesarean section.

There were several limitations to our study. First, we only studied the effect of dexmedetomidine combined with butorphanol on mothers and did not study the content of dexmedetomidine and butorphanol in maternal milk and whether they affected neonates. Second, the sample size included in this study is small, so the results may be biased to a certain extent. The results of this study need to be further verified by a systematic study with large sample size. At last, we only analyzed two doses of dexmedetomidine, and more doses of dexmedetomidine need to be studied to further explore the optimal dose for clinical application.

## Conclusion

Dexmedetomidine combined with butorphanol can improve the analgesic and sedative effects in continuous analgesia after a cesarean section, and the analgesic and sedative effects of dexmedetomidine in the high-dose group are better than those in the low-dose group.

## Data Availability

The original contributions presented in the study are included in the article/supplementary material; further inquiries can be directed to the corresponding author/s..

## References

[1] Gelman D Gelmanas A Urbanaitė D Tamošiunas R Sadauskas S Bilskiene D Role of multimodal analgesia in the evolving enhanced recovery after surgery pathways. Medicina. (2018) 54(2):20. 10.3390/medicina54020020 PMC6037254 30344251

[2] Wang J Yin Y Zhu Y Xu P Sun Z Miao C Thoracic epidural anaesthesia and analgesia ameliorates surgery-induced stress response and postoperative pain in patients undergoing radical oesophagectomy. J Int Med Res. (2019) 47(12):6160–70. 10.1177/0300060519866943 31426685 PMC7045687

[3] Kehlet H. Postoperative pain, analgesia, and recovery-bedfellows that cannot be ignored. Pain. (2018) 159(Suppl 1):S11–6. 10.1097/j.pain.0000000000001243 30113942

[4] Zieliński J Morawska-Kochman M Zatoński T. Pain assessment and management in children in the postoperative period: a review of the most commonly used postoperative pain assessment tools, new diagnostic methods and the latest guidelines for postoperative pain therapy in children. Adv Clin Exp Med. (2020) 29(3):365–74. 10.17219/acem/112600 32129952

[5] Chapman CR Vierck CJ. The transition of acute postoperative pain to chronic pain: an integrative overview of research on mechanisms. J Pain. (2017) 18(4):359.e1–9.e38. 10.1016/j.jpain.2016.11.004 27908839

[6] Glare P Aubrey KR Myles PS. Transition from acute to chronic pain after surgery. Lancet. (2019) 393(10180):1537–46. 10.1016/S0140-6736(19)30352-6 30983589

[7] Roofthooft E Joshi GP Rawal N Van de Velde M. PROSPECT working group of the European Society of Regional Anaesthesia and Pain Therapy and supported by the Obstetric Anaesthetists’ Association. PROSPECT guideline for elective caesarean section: updated systematic review and procedure-specific postoperative pain management recommendations. Anaesthesia. (2021) 76(5):665–80. 10.1111/anae.15339 33370462 PMC8048441

[8] Kintu A Abdulla S Lubikire A Nabukenya MT Igaga E Bulamba F Postoperative pain after cesarean section: assessment and management in a tertiary hospital in a low-income country. BMC Health Serv Res. (2019) 19(1):68. 10.1186/s12913-019-3911-x 30683083 PMC6347795

[9] Carr ZJ Cios TJ Potter KF Swick JT. Does dexmedetomidine ameliorate postoperative cognitive dysfunction? A brief review of the recent literature. Curr Neurol Neurosci Rep. (2018) 18(10):64. 10.1007/s11910-018-0873-z 30083844

[10] Keating GM. Dexmedetomidine: a review of its use for sedation in the intensive care setting. Drugs. (2015) 75(10):1119–30. 10.1007/s40265-015-0419-5 26063213

[11] Tasbihgou SR Barends CRM Absalom AR. The role of dexmedetomidine in neurosurgery. Best Pract Res Clin Anaesthesiol. (2021) 35(2):221–9. 10.1016/j.bpa.2020.10.002 34030806

[12] Ji J Lin W Vrudhula A Xi J Yeliseev A Grothusen JR Molecular interaction between butorphanol andκ-opioid receptor. Anesth Analg. (2020) 131(3):935–42. 10.1213/ANE.0000000000005017 32701545 PMC7668422

[13] Zhu Z Zhang W. Efficacy and safety of butorphanol use in patient-controlled analgesia: a meta-analysis. Evid Based Complement Alternat Med. (2021) 2021:5530441. 34335812 10.1155/2021/5530441 PMC8324365

[14] Ding X Luo Y Shi L Liu C Yan Z. Butorphanol in combination with dexmedetomidine provides efficient pain management in adult burn patients. Burns. (2021) 47(7):1594–601. 10.1016/j.burns.2020.12.025 33958243

[15] Ahsan MZ Khan FU Zhao MJ Wang YX. Synergistic interaction between butorphanol and dexmedetomidine in antinociception. Eur J Pharm Sci. (2020) 149:105322. 10.1016/j.ejps.2020.105322 32289662

[16] Beardsley PM Zhang Y. Synthetic opioids. Handb Exp Pharmacol. (2018) 252:353–81. 10.1007/164_2018_149 30242482

[17] Nedeljkovic SS Kett A Vallejo MC Horn JL Carvalho B Bao X Transversus abdominis plane block with liposomal bupivacaine for pain after cesarean delivery in a multicenter, randomized, double-blind, controlled trial. Anesth Analg. (2020) 131(6):1830–39. 10.1213/ANE.0000000000005075 32739962 PMC7643795

[18] Nie Y Liu Y Luo Q Huang S. Effect of dexmedetomidine combined with sufentanil for post-caesarean section intravenous analgesia: a randomised, placebo-controlled study. Eur J Anaesthesiol. (2014) 31(4):197–203. 10.1097/EJA.0000000000000011 24463478

[19] Imani F Rahimzadeh P Faiz HR Nowruzina S Shakeri A Ghahremani M. Comparison of the post-caesarean analgesic effect of adding dexmedetomidine to paracetamol and ketorolac: a randomized clinical trial. Anesth Pain Med. (2018) 8(5):e85311. 30538943 10.5812/aapm.85311 PMC6252045

[20] Liu S Peng P Hu Y Liu C Cao X Yang C The effectiveness and safety of intravenous dexmedetomidine of different concentrations combined with butorphanol for post-caesarean section analgesia: a randomized controlled trial. Drug Des Devel Ther. (2021) 15:689–98. 10.2147/DDDT.S287512 33628014 PMC7899314

[21] Jiang W Wang Q Xu M Li Y Yang R Song X Assessment of different loading doses of dexmedetomidine hydrochloride in preventing adverse reaction after combined spinal–epidural anaesthesia. Exp Ther Med. (2017) 13(6):2946–50. 10.3892/etm.2017.4335 28587365 PMC5450646

[22] Chen Z Tang R Zhang R Jiang Y Liu Y. Effects of dexmedetomidine administered for postoperative analgesia on sleep quality in patients undergoing abdominal hysterectomy. J Clin Anesth. (2017) 36:118–22. 10.1016/j.jclinane.2016.10.022 28183547

[23] Ide S Minami M Ishihara K Uhl GR Satoh M Sora I Abolished thermal and mechanical antinociception but retained visceral chemical antinociception induced by butorphanol in mu-opioid receptor knockout mice. Neuropharmacology. (2008) 54(8):1182–8. 10.1016/j.neuropharm.2008.03.008 18417173 PMC3769935

[24] Sanchez LC Elfenbein JR Robertson SA. Effect of acepromazine, butorphanol, or *N*-butylscopolammonium bromide on visceral and somatic nociception and duodenal motility in conscious horses. Am J Vet Res. (2008) 69(5):579–85. 10.2460/ajvr.69.5.579 18447787

[25] Waxman JA Shenouda KG Lin HS. Assessment and management of postoperative pain associated with sleep apnea surgery. Otolaryngol Clin North Am. (2020) 53(5):765–77. 10.1016/j.otc.2020.05.006 32564947

[26] Su X Wang DX. Improve postoperative sleep: what can we do? Curr Opin Anaesthesiol. (2018) 31(1):83–8. 10.1097/ACO.0000000000000538 29120927 PMC5768217

[27] Maduska AL Hajghassemali M. A double-blind comparison of butorphanol and meperidie in labour: maternal pain relief and effect on the newborn. Can Anaesth Soc J. (1978) 25(5):398–404. 10.1007/BF03006569 359109

[28] Fontaine GV Der Nigoghossian C Hamilton LA. Melatonin, ramelteon, suvorexant, and dexmedetomidine to promote sleep and prevent delirium in critically ill patients: a narrative review with practical applications. Crit Care Nurs Q. (2020) 43(2):232–50. 10.1097/CNQ.0000000000000304 32084065

[29] Huang X Lin D Sun Y Wu A Wei C. Effect of dexmedetomidine on postoperative sleep quality: a systematic review. Drug Des Devel Ther. (2021) 15:2161–70. 10.2147/DDDT.S304162 34045850 PMC8149279

[30] Zhang T Yu Y Zhang W Zhu J. Comparison of dexmedetomidine and sufentanil as adjuvants to local anesthetic for epidural labor analgesia: a randomized controlled trial. Drug Des Devel Ther. (2019) 13:1171–5. 10.2147/DDDT.S197431 31043770 PMC6469486

[31] Hernández-Avalos I Valverde A Ibancovichi-Camarillo JA Sánchez-Aparicio P Recillas-Morales S Osorio-Avalos J Clinical evaluation of postoperative analgesia, cardiorespiratory parameters and changes in liver and renal function tests of paracetamol compared to meloxicam and carprofen in dogs undergoing ovariohysterectomy. PLoS One. (2020) 15(2):e0223697. 10.1371/journal.pone.0223697 32059002 PMC7021320

[32] Lopes A Seligman Menezes M Antonio Moreira de Barros G. Chronic postoperative pain: ubiquitous and scarcely appraised: narrative review. Braz J Anesthesiol. (2021) 71(6):649–55. 34715995 10.1016/j.bjane.2020.10.014 PMC9373680

[33] Barnett T Denke L. Managing postoperative pain with opioid-sparing therapies. Nursing. (2020) 50(12):60–3. 10.1097/01.NURSE.0000694772.54730.b8 33497097

[34] McDonald DD Ward C Zhang Y. Development of the adverse analgesic drug event measure. Nurs Res. (2020) 69(4):299–306. 10.1097/NNR.0000000000000427 32084103

